# The patterns of family genetic risk scores for eleven major psychiatric and substance use disorders in a Swedish national sample

**DOI:** 10.1038/s41398-021-01454-z

**Published:** 2021-05-27

**Authors:** Kenneth S. Kendler, Henrik Ohlsson, Jan Sundquist, Kristina Sundquist

**Affiliations:** 1 Virginia Institute for Psychiatric and Behavioral Genetics, Virginia Commonwealth University, Richmond, VA USA; 2 Department of Psychiatry, Virginia Commonwealth University, Richmond, VA USA; 3 Center for Primary Health Care Research, Lund University, Malmö, Sweden; 4 Department of Family Medicine and Community Health, Department of Population Health Science and Policy, Icahn School of Medicine at Mount Sinai, New York, NY USA

**Keywords:** Diagnostic markers, Schizophrenia

## Abstract

To clarify the structure of genetic risks for 11 major psychiatric disorders, we calculated, from morbidity risks for disorders in 1st–5th degree relatives controlling for cohabitation effects, in the Swedish population born between 1932 and 1995 (*n* = 5,830,014), the family genetic risk scores (FGRS) for major depression (MD), anxiety disorders (AD), obsessive-compulsive disorder (OCD), bipolar disorder (BD), schizophrenia (SZ), bulimia (BUL), anorexia nervosa (AN), alcohol use disorder (AUD), drug use disorder (DUD), ADHD, and autism-spectrum disorder (ASD). For all affected individuals, we calculated their mean standardized FGRS for each disorder. The patterns of FGRS were quite similar for MD and AD, and for AUD and DUD, but substantially less similar for BUL and AN, BD and SZ, and ADHD and ASD. While OCD had high levels of FGRS for MD and AD, the overall FGRS profile differed considerably from MD and AD. ADHD FGRS scores were substantially elevated in AUD and DUD. FGRS scores for BD, OCD, AN, ASD, ADHD, and especially SZ were relatively disorder-specific while genetic risk for MD and AD had more generalized effects. The levels of FGRS for BMI, coronary artery disease, and educational attainment across our disorders replicated prior associations found using molecular genetic methods. All diagnostic categories examined had elevated FGRS for many disorders producing, for each condition, an informative FGRS profile. Using a novel method which approximates, from pedigree data, aggregate genetic risk, we have replicated and extended prior insights into the structure of genetic risk factors for key psychiatric illnesses.

## Introduction

Several different approaches have been taken to clarify the structure of the genetic risk factors for major psychiatric disorders (including substance use disorders). Most commonly, structural equation modeling (SEM) has been applied to twin samples^1–3^ or to polygenic risk scores (PRS) generated from genome-wide association studies^4,5^. These studies have been quite informative but have some limitations. For twin studies, sample sizes have not been insufficient to study key rarer disorders such as schizophrenia and autism spectrum disorder. For molecular genetic studies, case–control samples have been typically assembled from a wide variety of countries, using a range of ascertainment strategies and diagnostic approaches. Furthermore, few approaches to date have utilized a different analytic approach—calculating the average magnitude of genetic risk for each proband group for a wide array of disorders.

In this report, we address some of these prior limitations by examining individuals with a lifetime diagnosis of 11 psychiatric disorders ascertained through national registries in the Swedish population born between 1932 and 1995 (*n* = 5,830,014): major depression (MD), anxiety disorders (AD), obsessive-compulsive disorder (OCD), bipolar disorder (BD), schizophrenia (SZ), bulimia (BUL), anorexia nervosa (AN), alcohol use disorder (AUD), drug use disorder (DUD), ADHD, and autism spectrum disorder (ASD). Family genetic risk scores (FGRS) are calculated for each disorder from the weighted rates of that disorder in 1st through 5th degree relatives, controlling for cohabitation effects. We added *Family* in this term to clearly differentiate this statistic from PRS, as the FGRS derives its information not from molecular variants but from the phenotypes of a proband’s *family*. This method is complimentary to PRS for the analysis of psychiatric disorders. Its current main advantage is its availability for the entire population of Sweden, coupled with excellent medical and other national registry data. Its advantage over twin and twin-family analyses conducted by structural modeling is its use of a far wider range of relatives thereby providing substantially greater information about genetic risk.

First, we examine our FGRS by disorder, comparing the patterns of the 11 FGRS scores in each of the group of probands affected with our 11 disorders one at a time. That is, a proband in this study is defined as any individual who was registered in the relevant Swedish registries with one or more of these 11 disorders. This approach allows us to examine the degree to which the genetic vulnerability of individual disorders is largely the result of an elevated FGRS for one disorder, for a few conditions or for a wide range of disorders, and the similarity of the FGRS profile, across pairs or groups of disorders.

Second, we examine our results by FGRS, ranking the scores of each specific FGRS from highest to lowest across our 11 disorders. Examined in this way, we can see the relative diagnostic specificity of each FGRS.

Third, we validate our FGRS by examining their association with three non-psychiatric phenotypes previously associated in genetic epidemiologic or molecular genetics studies with some of our psychiatric disorders: body mass index (BMI), coronary artery disease (CAD), and years of education (YOE).

## Methods

**Fig. 1:**
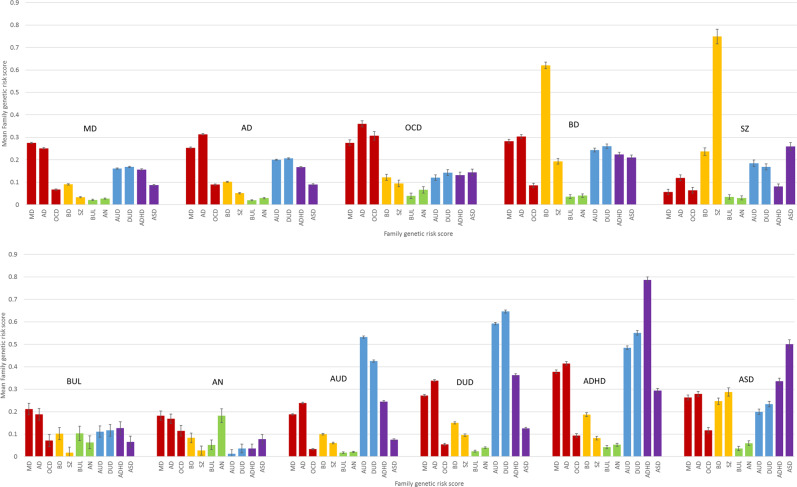
The mean Genetic Risk Score for 11 disorders in individuals diagnosed with those 11 disorders grouped by Disorder. The *y*-axis is the mean *z*-score for the genetic risk scores. The disorders are: major depression (MD), anxiety disorders (AD), obsessive-compulsive disorder (OCD), bipolar disorder (BD), schizophrenia (SZ), bulimia (BUL), anorexia nervosa (AN), alcohol use disorder (AUD), drug use disorder (DUD), ADHD, and autism-spectrum disorder (ASD). For clarity, we provide different color codes for the disorders divided into five categories of internalizing disorders (MD, AD, and OCD), psychotic disorders (BD and SZ), eating disorders (BUL and AN), substance use disorders (AUD and DUD), and neurodevelopmental disorders (ADHD and ASD).

We collected information on individuals from Swedish population-based registers with national coverage linking each person’s unique personal identification number which, for confidentiality, was replaced with a serial number by Statistics Sweden. This study was approved by the Regional Ethical Review Board of Lund (No. 2008/409, 2012/795, and 2016/679). Our database consisted of all individuals born in Sweden between 1932 and 1995 of

parents who themselves were born in Sweden and followed through Dec 31, 2017. We added the later requirement to ensure roughly comparable numbers of relatives for each of our probands. In the database, we included date of registration for major depression (MD), anxiety disorders (AD), obsessive-compulsive disorder (OCD), bipolar disorder (BD), schizophrenia (SZ), bulimia (BUL), anorexia nervosa (AN), alcohol use disorder (AUD), drug use disorder (DUD), ADHD, and autism spectrum disorder (ASD), utilizing ICD-8, 9, 10 codes from Swedish national primary care, specialist and hospital registries as well as information from Prescription and Criminal registers for AUD and DUD (see appendix Table 1 for full definitions). We also included individual genetic risk scores (FGRS) for all traits. For validation purposes, we also included FGRSs for BMI, CAD, and YOE. The FGRSs were based on selected 1st, 2nd, 3rd, 4th, and 5th degree relatives to the probands with a mean of 40.1 relatives per proband. Briefly (see appendix Fig. 1 for full details), we first calculated the morbid risk for the phenotype in our sample of relatives based on age at first registration and then we transformed the binary trait into an underlying liability distribution, with the threshold that divides the population into the two categories for the trait. Thereafter, we calculated the mean *z*-score for relatives with the trait and the mean *z*-score for individuals without. For 1st degree relatives we also multiplied the *z*-score with a factor that sought to correct for the influence of shared environmental factors separately for siblings and parent–offspring pairs. For parent–offspring pairs, this correction was implemented by comparing the resemblance, by logistic regression, for father–offspring pairs where the father sired and raised his child (that is, a father in an intact family) to the resemblance between children and their not-lived-with fathers, i.e., those who sired their offspring but never lived with or near them when they were growing up. We have examined such not-lived-with fathers in several prior extended adoption studies^6,7^ as reflecting parent–offspring resemblance resulting only from genetic effects, analogous to the biological parent in an adoption design. For sibling pairs, we compared the resemblance in half-sibs who were versus were not reared together. Having the same genetic relationship between these siblings, this comparison also isolates the genetic from the shared environmental effects. As seen in Fig. 1 in the appendix, the correction factors—the degree of the resemblance for our individual diagnoses that was retained after discounting the effect of shared environment—varied, across diagnoses, from 0.67 to 0.99 for parent–offspring pairs and from 0.52 to 0.88 for sibling pairs.

**Table 1:** Descriptive features of our population cohort and case samples.

Total, *n*	5,830,014		
Females, *n*	2,846,911 (48.8%)		
Males, *n*	2,983,103 (51.2%)		
Mean (SD) age at follow-up	54.4 (18.1)		
	Prevalence (%)		
	All	Females	Males
Major depression	11.4	14.7	8.2
Anxiety disorders	10.6	13.7	7.7
Obsessive-compulsive disorder	0.5	0.7	0.4
Bipolar disorder	1.2	1.4	0.9
Schizophrenia	0.5	0.4	0.5
Bulimia	0.1	0.2	0.01
Anorexia	0.2	0.4	0.02
Alcohol use disorder	6.2	3.4	8.8
Drug abuse	3.5	2.5	4.5
ADHD	1.4	1.2	1.5
Autism spectrum disorder	0.7	0.6	0.8

Within each type of relative, we then had two components—the sum of the *z*-score and the total weighted number of relatives. These two components were weighted according to the genetic resemblance to the proband. For each proband, we summed the two components across all groups of relatives and used the quotient between the two components. Finally, to obtain the individual FGRS, we multiplied the quotient with a shrinkage factor based on the variance of the *z*-score across all relatives, the variance in the mean *z*-score across all probands, and the number of weighted number of relatives for each proband. So that the FGRSs would be more comparable across traits and to reduce the effect of register coverage, we standardized the FGRS by year of birth into a *z*-score with mean = 0 and SD = 1.

For our first and second aims, we calculated the mean individual FGRSs for the 11 disorders among individuals affected, individually, with each of the 11 conditions. As a validation effort, we also examined the mean individual FGRSs for BMI, CAD, and YOE among individuals affected with our 11 disorders. All analyses were performed using SAS 9.4^8^.

## Results

### Sample description

Descriptive results for our sample are seen in Table 1. Our cohort included 5,830,014 individuals with a mean (SD) age at follow-up of 54.4 (18.1). The 11 disorders had lifetime prevalences ranging from 0.1% for BUL to 11.4% for MD. We observe the expected sex ratio with a female preponderance for MD, AD, OCD, BD, BUL, and AN, and a male preponderance for SZ, AUD, DUD, ADHD, and ASD.

### Analysis by disorder

Figure 1 presents, one by one, the 11 mean standardized FGRS ± 95% CIs for individuals affected with each of our 11 disorders. Of the many interesting patterns seen in this figure, we emphasize two. First, the general pattern of FGRS permits the division of our 11 disorders into 3 groups. For four disorders, BD, SZ, ADHD, and ASD, the FGRS scores for that disorder in affected individuals are substantially higher than that for any of the 10 other FGRS. For example, for individuals affected with SZ, the FGRS for SZ is more than three times higher than the next highest FGRS, in this case for ASD.

For four disorders, MD, AD, AUD, and DUD, their own FGRS are also highest in affected individuals but FGRS for one or more other disorders are nearly as high. For example, in individuals with MD, the mean AD FGRS score is nearly as high as the mean MD FGRS score. For subjects with DUD, the mean AUD FGRS is nearly as high as the DUD FGRS. For three disorders, OCD, BUL, and AD, the highest FGRS scores in affected individuals are *not* for the disorder themselves but instead are for MD for the two eating disorders and AD for OCD.

Second, by comparing the FGRS profile across disorders, we can observe several groups of disorders with important shared patterns of FGRS scores but often also with unique disorder-specific features. MD, AD, and OCD all share similarly elevated levels of MD and AD FGRS and the overall pattern of the other disorders is quite similar for MD and AD. OCD, by contrast, is more distinctive compared to the other two internalizing disorders, with a much higher FGRS for OCD, and also, compared with MD and AD, elevated levels of FGRS for SZ and ASD and reduced levels of FGRS for AUD and DUD. DUD and AUD have a robust reciprocal relationship where each disorder providing the second highest FGRS for the other disorder. However, DUD has higher levels for nearly every FGRS compared to AUD, particularly MD, AD, ADHD, and ASD. ADHD is a looser member of these two substance use disorders as the AUD and DUD FGRS comprise the second and third highest FGRS in ADHD subjects. SZ and ASD share some similarities in their profiles. ASD FGRS is the second higher FGRS for subjects with schizophrenia and the SZ FGRS is the third highest FGRS for individuals with ASD. Although both are eating disorders, the FGRS profiles for BUL and AN share some similarities but many differences. The highest FGRS for both disorders are for MD and AD. BUL has much higher levels of AUD, DUD, and ADHD FGRS than does AN, while AN has higher OCD FGRS. Furthermore, the FGRS for BUL is quite low in subjects with AN. Although both are often considered “psychotic disorders”, the FGRS profiles for BD and SZ are quite different. FGRS scores for MD, AD, and ADHD are considerably higher in the BD versus SZ subjects and the cross-loading of their own FGRS scores (that is levels of BD FGRS in SZ subjects and SZ FGRS in BD subjects) is much more modest than are seen with the MD–AD and AUD–DUD pairs.

### Analysis by genetic risk score

**Fig. 2:**
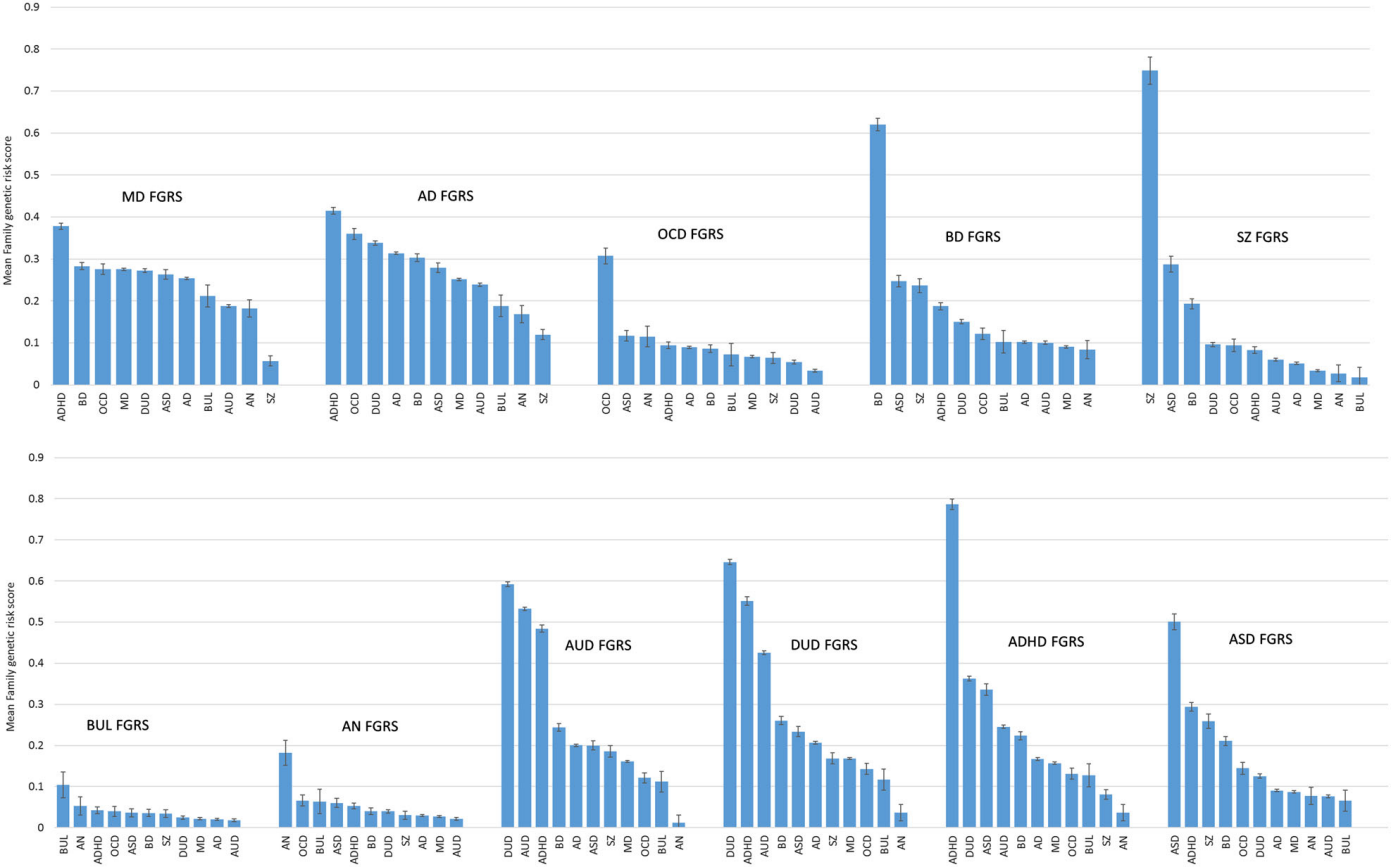
The mean genetic risk score for 11 disorders in individuals diagnosed with those 11 disorders grouped by genetic risk score. In this figure, the individual genetic risk scores are grouped by genetic risk score, from highest to lowest score, not by disorder as in Fig. 1. The *y*-axis is the mean *z*-score for the genetic risk scores. For initials of the disorders, see Fig. 1 legend.

Figure 2 presents the same data but arranged by FGRS rather than by disorder. For each FGRS, the disorders are ranked by FGRS from highest to lowest. We here emphasize two broad points from these results. First, we can examine the pattern of the specificity of each FGRS by two criteria: (i) is the FGRS scores highest in the disorder from which the FGRS is created? If yes, (ii) is there a substantial difference between the mean FGRS for that disorder and for the disorder with the second highest FGRS which we operationalize as a minimum 2:1 ratio? Eight of the 11 disorders meet the first criterion of specificity, all but MD, AD, and AUD. MD failed because subjects with ADHD have a slightly higher mean FGRS for MD than those with MD. AD fails because individuals with both ADHD and OCD have an AD FGRS slightly higher than do AD subjects. The next criterion was met by six of the eight disorders: OCD, BD, SZ, BUL, AN, and ADHD. DUD did not meet these conditions because of high levels of DUD FGRS in subjects with both ADHD and AUD. The ASD FGRS did not meet this criterion because of the relatively high levels of ASD FGRS in subjects with ADHD.

Second, the findings displayed by FGRS in Fig. 2 permit other observations of patterns of resemblance across disorders based on the relative levels of their particular FGRS scores. For example, patients with ASD had the second highest FGRS scores for BD and SZ and the third highest FGRS score for ADHD. Patients with ADHD had the second highest FGRS scores for ASD and DUD and the 3rd highest score for AUD. Aside from SZ itself, only two other disorders had appreciably increased risk for SZ FGRS, ASD and BD. Individuals with AN had the lowest FGRS scores for AUD, DUD, and ADHD and the second lowest for SZ and AD.

### Validation of FGRS scores

**Fig. 3:**
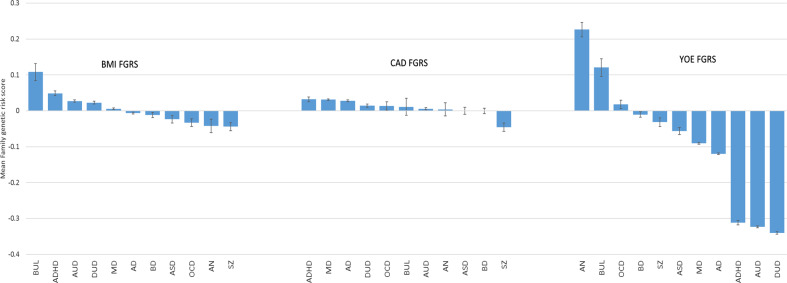
Validation of the FGRS Scores. The mean genetic risk score for body mass index (BMI), coronary artery disease (CAD), and years of education (YOE) in 11 disorders ascertained from the general Swedish population. The *y*-axis is the mean *z*-score for the genetic risk scores. The disorders are ranked from highest to lowest score.

Given the novelty of this method, our final analyses attempt to validate our FGRS method by examining, across our 11 disorders, the FGRS scores for BMI, CAD, and YOE (Fig. 3). For BMI, the effects were modest with the highest and lowest FGRS scores seen for BUL and AN, respectively. For CAD, the effects were even smaller, with AN and OCD having the highest mean risk and SZ the lowest. The effects were much larger for YOE where high YOE FGRS were seen, in order, for AN, BUL, OCD, BD, and ASD. Lower than average scores were seen, from largest to least reduction from the mean, for DUD, AUD, ADHD, AD, MD, and SZ.

## Discussion

Of the many points of possible interest in these results, we review four. *First*, a number of prior approaches, using latent variables applied to genetic correlations, have examined the structure of genetic risks for major psychiatric disorders^1–5^. Using a different method, our results also provide nuanced evidence for the inter-relationships of the genetic risks for different disorders. The two most striking examples are the close relationships revealed between MD and AD, and between AUD and DUD.

For MD and AD, consistent with prior evidence for a close genetic association between these two “internalizing disorders”^1,3,5^, the relationship is evidenced both by a reciprocal relationship between their FGRS scores (AD FGRS is the second highest score for MD patients and MD FGRS is the second highest FGRS for AD patients), and by a quite similar profile of their other FGRS scores. It is instructive to compare their two profiles with that of OCD which, when included in phenotypic analyses, typically aligns in factor analyses with other internalizing disorders^9,10^ but in a recent molecular genetic multivariate analysis it was shown to cluster instead with AN and Tourette’s syndrome^4^. With AD and MD, OCD shares high levels of AD and MD FGRS, but the pattern of other FGRS are quite different including, consistent with prior studies^4,11^, elevated levels of genetic risk for AN.

Congruent with prior evidence for shared genetic underpinnings for externalizing disorders^1,3,5^, the strong relationship between AUD and DUD is marked by sharing the same two highest FGRS for AUD and DUD. But differences in the FGRS profiles of these two drug use disorders are also evident. In particular, DUD, compared to AUD, has considerably higher levels of FGRS for MD, AD, BD, SZ, and ADHD.

However, our findings did not confirm close genetic relationships between two other pairs of disorders expected to be closely genetically related: AN and BUL, and BD and SZ. Family and twin studies show substantial sharing of genetic risk between AN and BUL^12,13^. However, the FGRS loadings for AN in BUL cases, and BUL in AN cases, were rather modest. Their genetic profiles also differed in other ways. For example, consistent with prior studies, BUL had a substantially higher FGRS scores for the externalizing disorders of AUD, DUD, and ADHD than did AN^14,15^.

Our results are also not consistent with recent reports of high genetic correlations between SZ and BD^16,17^, but are with earlier family studies^18–20^ showing quite modest co-aggregation. While the FGRS score of BD is the second highest observed for SZ, it is much weaker, and the association is not reciprocal as the SZ FGRS is the eighth strongest FGRS for BD. Furthermore, the profile for other FGRS scores differs meaningful across the two disorders.

Our results do provide support for a modest reciprocal genetic relationship between SZ and ASD reported elsewhere^17,21^ as the ASD FGRS is the second strongest for SZ and the SZ FGRS is the third strongest for ASD. Also, consistent with prior molecular genetics’ findings^4,5^, our two neurodevelopmental disorders have a reciprocal genetic inter-relationship as the ADHD FGRS is the second strongest for ASD, and ASD FGRS the third strongest for ADHD.

*Second*, the findings as presented in Fig. 2 help clarify the degree of disorder specificity of our 11 FGRS. This can be best illustrated by comparing the pattern of results for the FGRS for SZ and MD. The profile for SZ is dominated by a single substantial FGRS for SZ itself nearly three times greater higher than the next strongest FGRS, which is for ASD. By contrast, the curve for the MD FGRS is flatter with five disorders having MD FGRS scores only modestly lower than that seen for MD itself. A second sign of the relative non-specificity of risk to MD is that the MD FGRS is not highest for MD itself. The other FGRS with a similar pattern of non-specificity is AD. Our results suggest that these two classical internalizing disorders^22^ have a genetic substrate somewhat different from the other disorders examined. They substantially predispose to two disorders that are common, often disabling and are leading causes of world-wide disability^23^. However, these genetic risk factors also make important contributions to a wider range of disorders including BD, ADHD, and substance use and eating disorders.

*Third*, given the novelty of our FGRS methods, we sought to validate them in three non-psychiatric phenotypes to see if we could replicate previously observed genetic associations. We examined BMI because of intriguing evidence that genetic risk for AN was associated with constitutional thinness^24,25^. We replicated that finding showing a stronger inverse association of AN FGRS with BMI than any of the other disorders. Consistent with prior reports, we also found a negative association between our FGRS for BMI and FGRS for SZ^26^ and BD^25^, and a positive association between the BMI FGRS and ADHD^25^. We also showed, for the first time to our knowledge, a positive correlation between the FGRS for BMI and BN, consistent with the finding that a BMI polygene risk score predicted binge eating and purging in a general population sample^27^.

Prior genetic epidemiological and molecular genetic data have found an association between risk for MD and risk for CAD^28,29^. We also replicated those results. Finally, previous studies have found a positive genetic correlation between the genetic predisposition to high educational attainment and risk for ASD, BD, and AN^24,30,31^ and a negative genetic correlation with AUD^32^ and DUD^33^. We could reproduce all these associations. The ability of FGRS to consistently replicate a range of previously reported genetic correlations supports the validity of this method.

*Fourth*, perhaps the greatest contribution of these analyses to insightful prior work on the genetic relationships between disorders has been the value of examining, for our patient cohorts, not only their genetic risk for the disorder from which they suffer, but also the pattern of their genetic risks for multiple other disorders. Our results suggest that examining such genetic profiles, we can gain a richer understanding of the genetic substrate for our disorders and the genetic relationship across disorders.

### Limitations

These findings should be viewed in the context of eight potential methodological limitations. First, the validity of the FGRS score is dependent on the quality of the available diagnoses in the Swedish national registries which has been well demonstrated for SZ, BD, and OCD^34–37^. The validity of MD diagnoses is supported by its prevalence, sex ratio, sibling and twin correlations, and associated psychosocial risk factors^38,39^. Genetic epidemiological findings for AUD, DUD, and eating disorders in Sweden have been similar to those found in other samples^7,13,40–42^. We are unaware of attempts to validate Swedish diagnoses for ADHD, AN, ASD, or BUL.

Second, ASD had a usually low male-to-female ratio of 1.33:1. We therefore examined this ratio as a function of age at first registration (appendix Fig. 2). Of note, in those first diagnosed under age 10, the ratio was in the expected range: > 3:1.

Third, we did not attempt to account formally for assortative mating in our analyses. However, the impact of any such spousal concordance is accounted for in our analyses in that an individual’s FGRS is calculated from both maternal and paternal relatives.

Fourth, we did not correct for the fact that many individuals in the Swedish population would appear multiple times in our risk scores. They could, for example, be a proband if affected with one of our 11 disorders, and also be a sibling, parent, uncle or cousin if they had affected relatives. We do not expect this approach to produce biases in our FGRS scores, but our confidence intervals are likely to be modestly underestimated.

Fifth, our diagnoses required individuals to present for medical treatment, have a criminal contact related to AUD or DUD, abused prescription drugs or took specific pharmacological treatments for AUD. We are therefore likely to miss some mildly affected individuals and cannot rule out correlated treatment seeking in relatives as a potential confounder.

Sixth, in comparing results across a wide range of disorders, the question of diagnostic hierarches arises. We took a minimalist approach, only utilizing a single DSM-5 hierarchy^43^: individuals with both MD and BD are coded as BD. The other diagnostic pairing of concern was SZ and BD, where 14.0% of those with SZ also had a BD diagnosis and 5.6% with BD also had a SZ diagnosis. For those diagnoses, we developed a hierarchy and included that with overall changes in results (see appendix Table 2 and Fig. 3).

Seventh, our control for cohabitation effects, detailed in appendix Fig. 1 step 4, are based on large samples of fathers from intact families versus not-lived-with fathers^6^ and half-sibs reared together and apart^44^. Across disorders, shared environmental effects contributed a mean of 19.8 ± 5.8% to parent–offspring and 23.7 ± 3.4% to sibling–sibling resemblance, for which we corrected in our analyses. These effects are only approximate, and we did not control for environmental influences on more distant relatives. The aggregate impact of these correction on our FRGS, as seen in appendix Table 3, was quite modest.

Finally, to explore the stability of our FGRS, we examined cohort and geographical differences in all 11 of our FGRS scores in appendix Fig. 4a, b. Reassuringly, we found only modest effects of time and space on our FGRS.

## Conclusions

Prior efforts to investigate the structure of genetic risk factors for psychiatric disorders have largely relied on structural equation analyses of twin samples or polygenic risk scores derived from large, often diverse, case–control cohorts. Our approach is different and hopefully complementary, utilizing rates of psychiatric illness in extended pedigrees of 5.8 million Swedes. An analysis of the resulting patterns showed that all diagnostic categories had elevated FGRS for multiple disorders. These FGRS profiles provided important insights into the structure of the genetic substrates of our major psychiatric disorders in many, but not all instances, replicating patterns of genetic sharing found using other methods. We can be particularly confident in these replicated findings given the persuasive argument that obtaining similar results using different methods (i.e., “triangulation”), is of greater value than replicating findings using the same methods^45^.

## Supplementary information

Supplemental material.
