# Supplementary material for: The patterns of family genetic risk scores for eleven major psychiatric and substance use disorders in a Swedish national sample
Source: Transl Psychiatry. 2021 May 27;11:326. doi: 10.1038/s41398-021-01454-z (PMC8160183; doi:10.1038/s41398-021-01454-z)

APPENDIX

Table 1 - Definition of phenotypes

|  | Registers Used | Definition |
| --- | --- | --- |
| Major Depression (MD) | The Swedish Hospital Discharge Register (coverage 1973-2017); Outpatient Care Register (national coverage 2001-2017); Primary Care Registry (Partly coverage from 1999-2017) | ICD-8: 296.2, 298.0, 300.4; ICD-9: 296.2, 296.4, 298.0, 300.4; ICD-10: F32, F33.  **Note**: all individuals with a registration for BD were excluded. |
| Anxiety Disorder (AD) | The Swedish Hospital Discharge Register (coverage 1973-2017); Outpatient Care Register (national coverage 2001-2017); Primary Care Registry (Partly coverage from 1999-2017) | ICD-8: 300.0, 300.2 ; ICD-9: 300A, 300C; ICD-10: F40, F41 |
| Obsessive-Compulsive Disorder [OCD] | The Swedish Hospital Discharge Register (coverage 1973-2017); Outpatient Care Register (national coverage 2001-2017); Primary Care Registry (Partly coverage from 1999-2017) | ICD-9: 300D; ICD-10: F42 |
| Bipolar Disorder (BD) | The Swedish Hospital Discharge Register (coverage 1973-2017); Outpatient Care Register (national coverage 2001-2017); Primary Care Registry (Partly coverage from 1999-2017) | ICD-8: 296.1, 296.3, 296.8, 296.9, 298.1; ICD-9: 296A, 296C, 296D, 296E, 296W, 298B; ICD-10: F30, F31 |
| Schizophrenia (SZ) | The Swedish Hospital Discharge Register (coverage 1973-2017); Outpatient Care Register (national coverage 2001-2017); Primary Care Registry (Partly coverage from 1999-2017) | ICD-8: 295.1, 295.2, 2953, 295.9, 295.6; ICD-9: 295B, 295C, 295D, 295G, 295X; ICD-10: F200, F201, F202, F203, F205, F209 |
| Bulimia (BUL) | The Swedish Hospital Discharge Register (coverage 1973-2017); Outpatient Care Register (national coverage 2001-2017); Primary Care Registry (Partly coverage from 1999-2017) | ICD-10: F502, F503 |
| Anorexia Nervosa (AN) | The Swedish Hospital Discharge Register (coverage 1973-2017); Outpatient Care Register (national coverage 2001-2017); Primary Care Registry (Partly coverage from 1999-2017) | ICD-9: 307B; ICD-10: F500 |
| Alcohol Use Disorder (AUD) | The Swedish Hospital Discharge Register (coverage 1973-2017); Outpatient Care Register (national coverage 2001-2017); Primary Care Registry (Partly coverage from 1999-2017); the Swedish Drug Register (2005-2017); the Swedish Mortality Register, and the Swedish Criminal Register (1973-2017) and the Swedish Suspicion Register (1998-2017) | Alcohol Use Disorder (AUD) was identified in the Swedish medical and mortality registries by ICD codes: ICD9: V79B, 305A, 357F, 571A-D, 425F, 535D, 291, 303, 980; ICD 10: E244, G312, G621, G721, I426, K292, K70, K852, K860, O354, T51, F10); in the Crime Register by codes 3005, 3201, which reflect crimes related to alcohol abuse; in the Suspicion Register by codes 0004, 0005 (Only those individuals with at least two alcohol-related crimes or suspicion of crimes from both Crime Register and Suspicion Register were included); in the Prescribed Drug Register by the drugs disulfiram (Anatomical Therapeutic Chemical (ATC) Classification System N07BB01), acamprosate (N07BB03), and naltrexone (N07BB04). |
| Drug Use Disorder (DUD) | The Swedish Hospital Discharge Register (coverage 1973-2017); Outpatient Care Register (national coverage 2001-2017); Primary Care Registry (Partly coverage from 1999-2017); the Swedish Drug Register (2005-2017); the Swedish Mortality Register, and the Swedish Criminal Register (1973-2017) and the Swedish Suspicion Register (1998-2017) | Drug abuse (DA) was identified in the Swedish medical and mortality registries by ICD codes (ICD8: Drug dependence (304); ICD9: Drug psychoses (292) and Drug dependence (304); ICD10: Mental and behavioral disorders due to psychoactive substance use (F10-F19), except those due to alcohol (F10) or tobacco (F17)); in the Suspicion Register by codes 3070, 5010, 5011, and 5012, that reflect crimes related to DA; and in the Crime Register by references to laws covering narcotics (law 1968:64, paragraph 1, point 6) and drug-related driving offences (law 1951:649, paragraph 4, subsection 2 and paragraph 4A, subsection 2). DA was identified in individuals (excluding those suffering from cancer) in the Prescribed Drug Register who had retrieved (in average) more than four defined daily doses a day for 12 months from either of Hypnotics and Sedatives (Anatomical Therapeutic Chemical (ATC) Classification System N05C and N05BA) or Opioids (ATC: N02A). |
| ADHD | The Swedish Hospital Discharge Register (coverage 1973-2017); Outpatient Care Register (national coverage 2001-2017); Primary Care Registry (Partly coverage from 1999-2017) | ICD-9: 314; ICD-10: F90 |
| Autism spectrum disorder (ASD) | The Swedish Hospital Discharge Register (coverage 1973-2017); Outpatient Care Register (national coverage 2001-2017); Primary Care Registry (Partly coverage from 1999-2017) | ICD-9: 299; ICD-10: F840, F841, F845, F849 |
| Body-mass index (BMI) | The Swedish conscript register for all males aged 18-19 who enlisted for military service between 1968 and 2005. During that time, Swedish law required all male citizens to enlist, with exceptions for those who were imprisoned or had severe chronic somatic or mental conditions or functional disabilities documented by a medical certificate (approximately 2%–3% annually). | BMI calculated from length and height in the Swedish Conscript register |
| Coronary artery disease (CAD) | The Swedish Hospital Discharge Register (coverage 1973-2017); Outpatient Care Register (national coverage 2001-2017); Primary Care Registry (Partly coverage from 1999-2017) | ICD-8: 410-414; ICD-9: 410-414; ICD-10: I20-I25 |
| Years of education (YOE) | The longitudinal integration database for health insurance and labor market studies (LISA) from 1990-2017 and The Swedish Census from 1970. | Highest achieved education measured in 1-7 levels that in turn were translated into number of years of education and then standardized with mean 0 and SD 1 by gender and year of birth. Note: In the analysis the Z-score is reversed so that higher values indicate lower education. In the registers the variable are as follows:  1-Pre-high school (7 years)  2-High School (9 years)  3-Upper Secondary School (11 years)  4-Upper Secondary School (12 years)  5-Post-secondary education (14 years)  6-Post-secondary education (17 years)  7- PhD education (21 years) |

**Figure 1 - Flow chart for calculation of the Genetic Risk Score (FFGRS):**

Step 2

Step 1

Information on relatives: Year of birth, sex, age at first registration for all traits, age at end of follow-up (2017-12-31 or age at death, age at emigration whichever came first)

Relative: 1^st^, 2^nd^, 3^rd^, 4^th^, and 5^th^ degree relatives to the probands. The mean (SD) number of proband were as follows: 1^st^ 4.55 (2.1); 2^nd^ 7.63 (4.5); 3^rd^ 8.61 (6.7); 4^th^ 12.44 (10.2); 5^th^ 6.90 (6.3). For 1^st^ degree relatives, we considered parents, children and full siblings. For 2^nd^ degree relatives, we considered aunts/uncles, grandparents, half-siblings, double first-cousins, grandchildren and nieces/nephews. For 3^rd^ degree relatives, we considered first cousins, grand aunts/uncles, aunts/uncles based on half-siblings to parent, nieces/nephews based on half-siblings, and grandchildren to full siblings. For 4^th^ degree relatives, we examined cousins based on half-siblings to parents, grand aunts/uncles based on half sibling to grandparent, first cousin once removed. For 5^th^ degree, relatives we examined children to grand aunts/uncles based on half-siblings and first cousin once removed based on half siblings.

Proband: All individuals born 1932-1995 in Sweden to Swedish born parents

Calculate the morbid risk for the trait by using age at first registration for traits among relatives

Use the distribution of age at first registration to weight relatives. The morbid risk followed a normal distribution with weights reflecting the proportion of risk period they had completed starting at age 15. All relatives registered for the specific trait were weighted 1 regardless of age

Registrations for traits are only available from 1973 and onwards, suggesting that relatives at older age do not have the possibility to be registered for the traits at younger ages. Therefore, we moved the weighting scale for each year for relatives born prior to 1958. This means that the risk period started at age 16 for relatives born 1957, at age 17 for relatives born 1956 and so on.

Step 2.1

Transform the binary variable (trait yes/no) into a z-score based on the threshold for each trait. We used separate thresholds for each decade of birth and sex.

Step 3

Step 4

Step 3.2

Apply the mean z-score among individuals above the threshold to all relatives with an registration for the traits and the mean z-score below the threshold for relatives without the trait

Calculate mean z-score for individuals above the threshold and for individuals below the threshold (within each decade of birth and sex). This was done by assigning individuals a z-score from a normal distribution and then calculate the mean z-score for all individuals above/below the threshold.

Step 3.1

Calculate the environmental correction parameter. For parent-offspring pairs, this was calculated by comparing the resemblance, by logistic regression, for father-offspring pairs where the father sired and raised his child to the correlations observed between children and their not-lived-with father who sired them but never lived with or near them when they were growing up. For sibling pairs, we compared the resemblance in half-sibs who were versus were not reared together. See table below for factor used for the different traits:

|  | Parent/Children | Siblings |
| --- | --- | --- |
| MD | 0.80 | 0.85 |
| AD | 0.87 | 0.81 |
| OCD | 0.79 | 0.74 |
| BD | 0.67 | 0.77 |
| SZ | 0.93 | 0.84 |
| BUL | * | 0.87 |
| AN | * | 0.88 |
| AUD | 0.99 | 0.69 |
| DA | 0.92 | 0.52 |
| ADHD | 0.42 | 0.81 |
| ASD | 0.83 | 0.61 |
| BMI | 0.90 | 0.66 |
| CAD | 0.83 | 0.70 |
| YOE | 0.82 | 0.73 |
| *No reliable estimate due to very low prevalence rates in males – we therefore used the mean among all other traits | | |

Step 8

Correct for the number of relatives. We multiplied the risk score with a shrinkage factor (SF) used in multilevel models based on (A) the variance of the z-score of the trait across all relatives, (B) the variance in the mean z-score across all probands, and (C) the number of weighted number of relatives for each proband. The SF is calculated as B / (B+A/C) and produces more shrinkage if B and C are small and A is large.

Average the relative-specific risk score across all relatives to a proband

Step 7

Step 6

Calculate the genetic risk score for each specific relative:

Z-score * weights reflecting the proportion of risk period they had completed * environmental correction * genetic resemblance

Step 5

Correct for difference by year of birth and county differences. There are 21 counties in Sweden. For each proband we used the county they had resided in during the maximum number of years (measured from 1969 and onwards) We standardized the risk score by year of birth and county of the proband into a z-score with mean 0 and SD 1. This was then used as the FGRS in the analyses.

Figure 2 – Male to Female Ratio of Cases of Autism Spectrum Disorder in the Swedish Registries as a Function of Age at First Registration

Table 2 Hierarchy for individuals with a registration of Schizophrenia and Bipolar Disorder

The table below illustrates how individuals were categorized when they had at least one registration for BD and at least one registration for SZ.

|  |  | Number of lifetime SZ diagnoses in the registers | | | | |
| --- | --- | --- | --- | --- | --- | --- |
|  |  | 1 (Group 1) | 2 (Group 2) | 3-5 (Group 3) | 6-10 (Group 4) | More than 10 (Group 5) |
| Number of lifetime BD diagnoses in the registers | 1 (Group 1) | Last diagnosis | Last diagnosis | Most common diagnosis | Most common diagnosis | Most common diagnosis |
|  | 2 (Group 2) | Last diagnosis | Majority of last 3 diagnoses | Majority of last 3 diagnoses | Most common diagnosis | Most common diagnosis |
|  | 3-5 (Group 3) | Most common diagnosis | Majority of last 3 diagnoses | Majority of last 3 diagnoses | Majority of last 3 diagnoses | Majority of last 5 diagnoses |
|  | 6-10 (Group 4) | Most common diagnosis | Most common diagnosis | Majority of last 3 diagnoses | Majority of last 5 diagnoses | Majority of last 5 diagnoses |
|  | More than 10 (Group 5) | Most common diagnosis | Most common diagnosis | Majority of last 5 diagnoses | Majority of last 5 diagnoses | Majority of last 5 diagnoses |

Figure 3 – Figure 1 From the Manuscript Redone with a Hierarchy Imposed between Schizophrenia and Bipolar Disorder
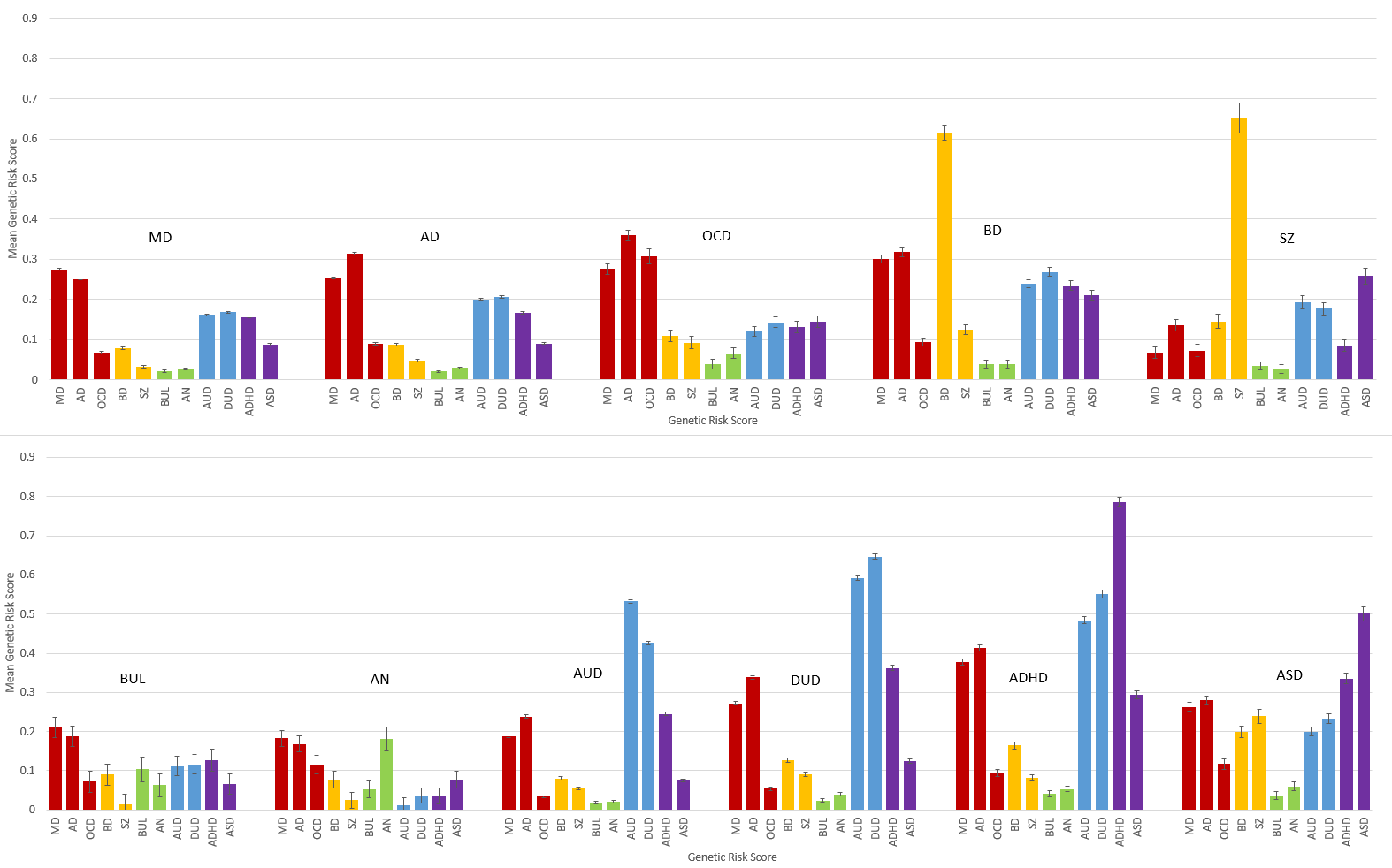


| Table 3 – Correlations between the Final FGRS and the FRGS Calculated When Dropping Major Steps in the Calculation | | | | | | | |
| --- | --- | --- | --- | --- | --- | --- | --- |
|  | MD FGRS | DUD FGRS | BP FGRS | SZ FGRS | AUD FGRS | SA FGRS | SD FGRS |
| **Correlation with the FGRS used in the ms for selected FGRS** | | | |  |  |  |  |
| FGRS(a) - 1^st^ degree relatives | 0.760 | 0.763 | 0.763 | 0.748 | 0.812 | 0.731 | 0.745 |
| FGRS(b) - no age correction | 0.949 | 0.995 | 0.992 | 0.987 | 0.806 | 0.999 | 0.982 |
| FGRS(c) - no cohabitation correction | 0.961 | 0.980 | 0.988 | 0.998 | 0.992 | 0.991 | 0.988 |
| FGRS(d) - no weighting for # relatives | 0.912 | 0.971 | 0.960 | 0.966 | 0.952 | 0.974 | 0.952 |
| FGRS(e) -std by YoB only | 0.973 | 0.990 | 0.994 | 0.990 | 0.992 | 0.983 | 0.996 |
| FGRS(f) - std by geography only | 0.955 | 0.951 | 0.952 | 0.923 | 0.969 | 0.948 | 0.952 |
| FGRS(g) - std only by entire sample | 0.935 | 0.946 | 0.948 | 0.919 | 0.962 | 0.934 | 0.950 |
|  |  |  |  |  |  |  |  |

Figure 4a - Stability of FGRS Scores for Geographical Region (North and South) within Sweden


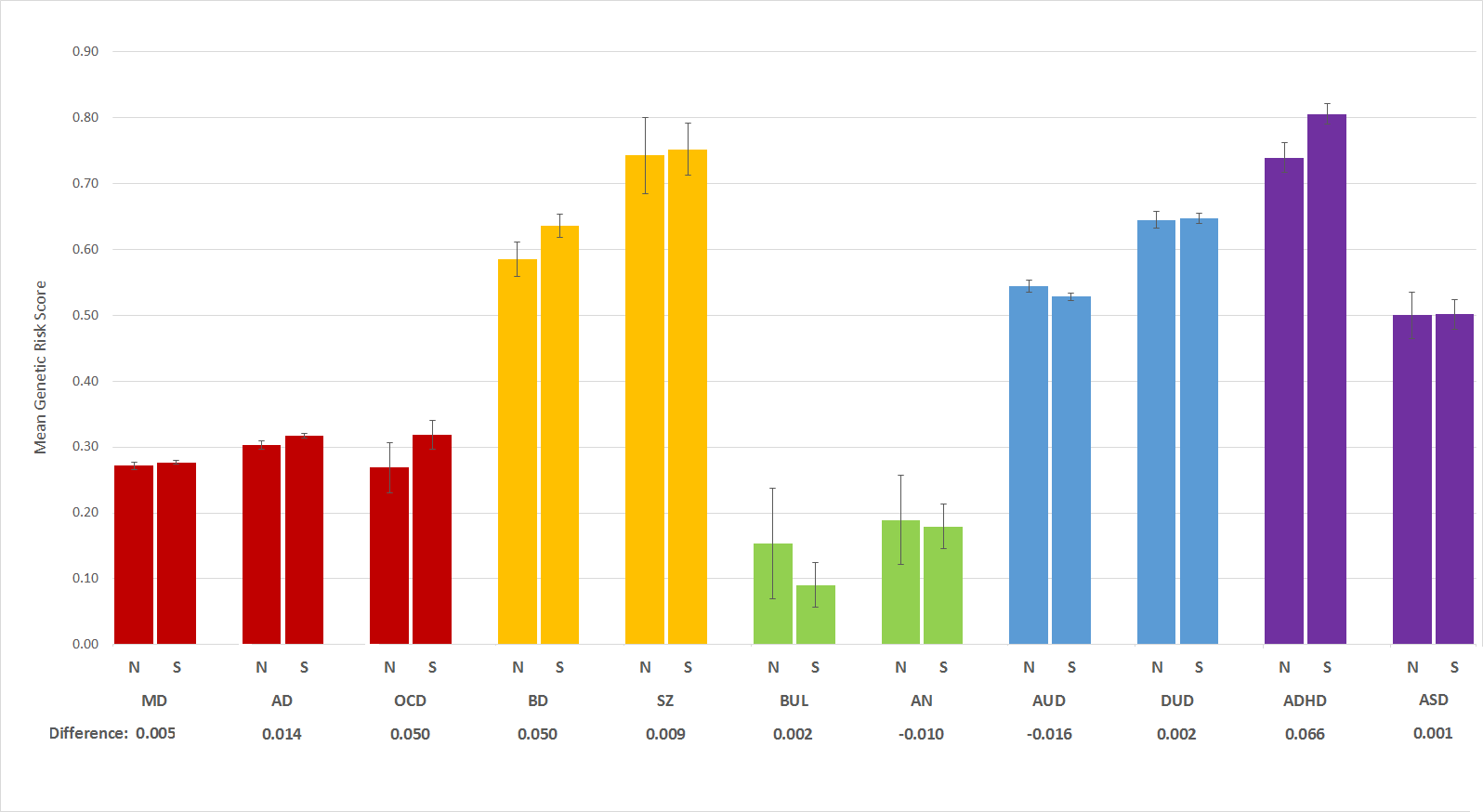


Figure 4b - Stability of FGRS Scores for Median split for Cohort (Old: 1932-1963 vs Young: 1964-1995)


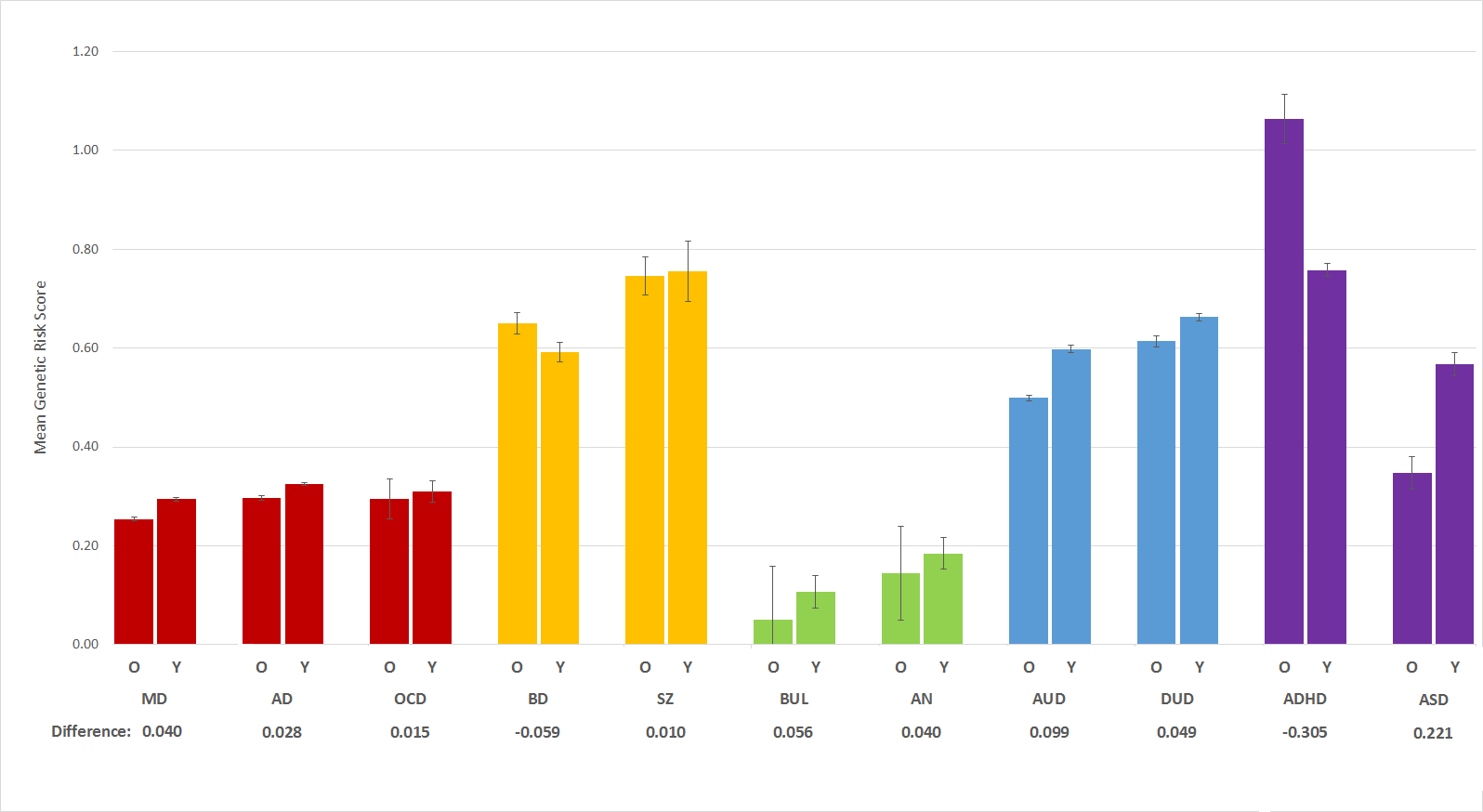

Supplement: Supplementary file 1 — Supplemental material. [file 41398_2021_1454_MOESM1_ESM.docx]
